# Neurological Symptoms and Their Associations With Inflammatory Biomarkers in the Chronic Phase Following Traumatic Brain Injuries

**DOI:** 10.3389/fpsyt.2022.895852

**Published:** 2022-06-24

**Authors:** Gangqin Li, Hao Liu, Yong He, Zeqing Hu, Yan Gu, Yan Li, Yi Ye, Junmei Hu

**Affiliations:** ^1^Department of Forensic Psychiatry, West China School of Basic Medical Sciences and Forensic Medicine, Sichuan University, Chengdu, China; ^2^West China School of Basic Medical Sciences and Forensic Medicine, Sichuan University, Chengdu, China; ^3^Department of Forensic Toxicological Analysis, West China School of Basic Medical Sciences and Forensic Medicine, Sichuan University, Chengdu, China

**Keywords:** traumatic brain injury, interleukin, chronic inflammation, neuropsychiatric disability, sleep disorder, headache

## Abstract

**Background:**

The underlying biological mechanisms for neurological symptoms following a traumatic brain injury (TBI) remain poorly understood. This study investigated the associations between serum inflammatory biomarkers and neurological symptoms in the chronic phase following moderate to severe TBI.

**Methods:**

The serum interleukin [IL]-1β, IL-4, IL-5, IL-6, IL-7, IL-8, IL-10, IL-12p70, and the tumor necrosis factor [TNF]-α in 72 TBI patients 6 months to 2 years post injury were measured. Neurological symptoms including depression, chronic headache, sleep disturbance, irritability, anxiety, and global neurological disability was assessed. The associations between the biomarkers and the neurological symptoms were assessed using correlation and regression analysis.

**Results:**

It was found that the most common post-injury symptom was sleep disturbance (84.7%), followed by chronic headaches (59.7%), irritability (55.6%), and depression (54.2%). TNF-α was a protective factor for chronic headache (OR = 0.473, 95% CI = 0.235–0.952). IL-6 was positively associated with sleep disturbance (r = 0.274, *p* = 0.021), while IL-5 and IL-12p70 were negatively associated with the degree of global neurological disability (r = −0.325, *p* = 0.006; r = −0.319, *p* = 0.007).

**Conclusion:**

This study provides preliminary evidence for the association between chronic inflammation with neurological symptoms following a TBI, which suggests that anti-inflammatory could be a potential target for post-TBI neurological rehabilitation. Further research with larger sample sizes and more related biomarkers are still needed, however, to elucidate the inflammatory mechanisms for this association.

## Introduction

Traumatic brain injuries (TBIs) are one of the leading causes of death and disability worldwide (1), with around 5.3 million people in the United States and nearly 7.7 million people in Europe living with a TBI-related disability (2). Post-TBI neurological symptoms, such as poor executive function, impaired attention, depression, sleep disturbance, chronic headaches, emotional dysregulation, poor decision-making, and aggressive behavior (3–5), can be a heavy burden on individuals, families, and society. TBIs are often described as a “silent epidemic” because the patients and family members know how devastating the effects can be; however, awareness is low and research into TBI-related neurological symptoms has been sparse, with the underlying mechanisms still being largely unknown (1).

Inflammation is an important secondary injury mechanisms after TBI (6) and has been reported to be associated with many neurological symptoms, such as depression, sleep disturbance, and headaches. Many studies have documented the associations between depression and immune dysregulation (7–9), and it has been found that higher concentrations of pro-inflammatory cytokines can directly contribute to the development of depressive symptoms (10). Dowlati et al. found that TNF-α and IL-6 concentrations were significantly higher in patients with depression compared with healthy control subjects, and suggested that depression was accompanied by an activation of the inflammatory response system (11). Review studies have also found that inflammation, and especially neuroinflammation, was one of the mechanisms for chronic post-traumatic headaches (12–14). Moreover, multiple line of evidence showed a reciprocal connection between the immune system and sleep, which suggested that the dysregulation of inflammation could be contributing to sleep disturbances, and vice versa (15, 16). Specifically, Irwin et al. reported that sleep disturbance was associated with increases in systemic inflammation (higher levels of CRP and IL-6) (16).

Based on these surmised associations between inflammation, TBI, and neurological symptoms, it is plausible to suggest that inflammation may be connected with post-TBI neurological disabilities. Several studies have investigated the associations between acute inflammation with the long-term outcome following TBI. For example, Nwachuku et al. reported that the cerebrospinal fluid (CSF) concentrations of inflammatory biomarkers (IL-1, IL-6, TNF-α, IFN-γ, IL-12p70, IL-10, and IL-8) in the first 5 days after a severe TBI had a negative association with the 6-month Glasgow Outcome Scale (GOS) scores (17). Juengst et al. found that higher levels of acute CSF cytokine surface markers (sVCAM-1, sICAM-1, and sFAS) were associated with a 3.92-fold increase in the odds of developing depression 6 months after a moderate to severe TBI (18). Kumar et al. reported that IL-1β, IL-6, IL-8, and IL-10 serum levels, and TNF-α were elevated 3 months after a severe TBI, and increased cytokine was, respectively, associated with 1.21 and 1.18 increases in the odds of an unfavorable GOS score at 6 and 12 months post-TBI (19). Similarly, Devoto et al. reported that acute inflammation after a TBI was related to chronic behavioral and neurological symptoms in military personnel (20). These results, therefore, preliminarily linked acute inflammation to long-term post-TBI outcomes; however, it is not clear whether chronic neurological and neurological symptoms in TBI patients are related to chronic inflammation is not clear.

Recent years, emerging researchers proposed that the acute post -TBI inflammation may transit from an adaptive immune response to a chronic, maladaptive response, leading to post-trauma depression and more significant disability (21–23). Several studies have reported that chronic neuroinflammation may exist long-term after a central nervous system (CNS) injury. For example, Ramlackhansingh found that increased microglial activation can be present up to 17 years post-TBI (24), and Johnson et al. found persistent inflammation and ongoing white matter degeneration up to 18 years after a single TBI (25). Studies have also found long-term microglial activation in TBI animals, particularly in subcortical areas remote from the focal injury (26), which was also evident in a postmortem study in people (27). Moreover, Witcher et al. found that microglia activation promotes persistent neuropathology and long-term functional impairments in neuronal homeostasis after TBI (28). On the other side, animal studies demonstrated that microglial depletion during chronic phase of experimental TBI reduced neurodegeneration and neurological deficits, such as cognitive and memory impairments (29–32). These findings provide preliminary evidence that neuroinflammation may persist for years and cause negative outcomes after a TBI; however, whether the systemic inflammation in the long-term phase post-TBI is associated with neurological symptoms is still unclear and needs further study.

To explore the neurological symptoms in the chronic post-TBI phase and their relationship with systemic inflammatory biomarkers, this study assessed the serum cytokines in patients with neurological symptoms more than 6 months after a moderate to severe TBI. The common neurological symptoms and global functions were evaluated using psychological scales. This study's exploration of the inflammation basis for neurological symptoms following a TBI could provide guidance on the provision of treatment and rehabilitation for people with chronic TBI-related neurological disabilities.

## Materials and Methods

### Participants and Biological Sample Collection/Processing

TBI patients referred for forensic evaluations for their neurological disability from May 2019 to May 2020 in a forensic center in Sichuan Province, China, were invited to participate. Seventy-two TBI patients were enrolled in the current study. All participants were of Chinese Han ethnicity, the average age was 47.7 ± 12.0 years, and there were 58 males (80.6%, average age was 46.6 ± 12.5 years) and 14 females (19.4%, average age was 52.5 ± 9.1 years). Eighty-nine percent of the injuries had been the result of a traffic accident, while the remaining had been the result of falls. The time interval from the injury to the forensic assessment ranged from 182 to 718 days, with the average interval being 334 ± 163 days (details are presented in Table 1). The enrollment criteria for participants were as follows: (a) 18–60 years old; (b) had a moderate to severe TBI indicated by symptoms and a head computed tomographic scan or magnetic resonance image; and (c) had neurological complaints for more than 6 months post-TBI. Participants were excluded if they: (a) had a cold in the previous month; (b) had a history of an inflammatory disorder, such as rheumatoid arthritis, multiple sclerosis, systemic lupus erythematosus and so on, because patients with these inflammatory diseases may have elevation of systemic inflammatory biomarkers (33–35), which would preclude the independent analysis of TBI-related inflammation; (c) had bleeding disorder because the venipuncture was used in this study; (d) have a history of cigarette smoking in the previous 6 months, because studies indicated that cigarette smoke may induce chronic inflammation and increase expression of inflammatory cytokines, such as IL-6, IL-8 and TNF-α (36–38); (e) had pre-injury neurological or psychiatric disorders (headache, anxiety, depression, sleep disturbance, intelligence deficits and so on), because these pre-injury symptoms may contribute to more severe neuropsychiatric symptoms after traumatic injury (39–41), which would influence the evaluation of the post-injury neurological symptoms; (f) had been dosed with anti-inflammatory or antidepressant drugs in the previous month. This study was approved by the Medical Ethics Committee of Sichuan University and written informed consent was obtained from all participants and their legal guardians.

**Table 1:** Demographic and injury data.

**Variables**	**Number (*%*)**	**Mean ±SD (range)**
Age, years	72 (100.0)	47.7 ± 12.0
Male	58 (80.6)	46.6 ± 12.5
Female	14 (19.4)	52.5 ± 9.1
**Injury type**		
Falls	8 (11.1)	
Motor vehicle/motorcycle accident	64 (88.9)	
Intervals post-injury, days		334 ± 163 (182–718)
180–365 days	44 (61.1)	
366–730 days	28 (38.9)	

Peripheral blood samples (2 ml) were collected in sterile tubes with EDTA-Na2 anticoagulants by standard venipuncture from each participant, after which they were centrifuged (3,000 rpm, 15 min) to extract the serum. The serum was then aliquoted and stored at −80°C until the batch analysis. There were nine inflammatory markers measured in the serum: IL-1β, IL-4, IL-5, IL-6, IL-7, IL-8, IL-10, IL-12p70, and TNF-α. The serum levels of these biomarkers were measured using a commercially available electrochemiluminescence immunoassay named U-PLEX Biomarker Group 1 (Human) Multiplex Assay (K15067L-1) (Meso Scale Discovery, Maryland, United States) according to the manufacturer's instructions (https://www.mesoscale.com/~/media/files/product%20inserts/u−plex%20biomarker%20group%201%20human%20insert−multiplex.pdf).A MESO QuickPlex SQ 120 machine and MSD Discovery Workbench software version 4.0.12 were respectively used for the detection and data analysis.

### Neurological Symptom Measures

#### Depression

Depression was assessed based on the International Classification of Diseases-10th revision (ICD-10) criteria, with the Patient Health Questionnaire-9 (PHQ-9) (42) employed to quantify the depression severity. The PHQ-9 comprises nine items that describe depressive symptoms, with participants required to rate their feelings in the previous 2 weeks on a 4-point Likert scale from 0 = “not at all” to 3 = “nearly every day.” The total score ranges from 0 to 27, with higher scores indicating increased symptom severity. We used a cut-off score of 5 and above for depression in Chinese population (43).

#### Anxiety

The Hamilton Anxiety Scale (HAMA) (44) was used to evaluate anxiety severity, which comprises 14 symptom-defined items. Participants were required to rate their feelings on a basic numeric scoring from 0 (not at all) to 4 (severe). The total scale score ranges from 0 to 56, with a cut-off score of 14 and above indicating anxiety (45), and higher score indicating higher severity.

#### Sleep Disturbance

The Pittsburgh sleep quality index (PSQI) was used to measure sleep quality and sleep patterns in the previous month, which analyzes seven factors: subjective sleep quality; sleep latency; sleep duration; habitual sleep efficiency; sleep disturbances; use of sleeping medications; and daytime dysfunction (46). The questions are rated on a 4-point Likert scale (0–3), with the score for each component then added to give a total score ranging from 0 to 21, with higher scores indicating poorer sleep quality. We created a categorical measure using a cut-off of 6 and above for sleep disturbance (47).

#### Irritability

Irritability was measured with 7 items derived from the Neuropsychiatry Inventory (NPI) (48). The items are yes/no questions about behavioral manifestations of irritability. Patients' caregivers were required to rate the frequencies (1–4, 1 = occasionally, 2 = often, 3 = frequently, 4 = very frequently) and severity (1–3, 1 = mild, 2 = moderate, 3 = severe) of the irritability symptoms is. The total score is the frequency score multiplied by the severity score, which ranges from 1 to 12, with higher scores indicating higher severity. This inventory has been validated in Chinese and TBI populations (49, 50).

#### Chronic Headache and IQ

Chronic headache was rated using a visual analog scale (score ranges from 0 to 100, higher scores indicate higher severity). Global intelligence was measured using the Chinese version of Wechsler Adult Intelligence Scale Revised (WAIS-RC, higher scores indicate higher IQ) (51).

#### Neurological Disability

Global neurological disability was evaluated based on the *Classification of Severity of Disability by Physical Injuries (National standard issued by the Supreme People's Court, the Supreme People's Procuratorate, the Ministry of Public Security, 2016)* criteria. The neurological disability degree ranges from 1 to 10, in which 10 is the lowest and 1 is the highest degree of disability. Multiple aspects are considered in the evaluation; intelligence, memory, concentration, cognition, depression, psychotic symptoms, daily life function, and vocational and social functions; which are similar to the factors in the Glasgow Outcome Scale-Extended (52) and the International Classification of Functioning, Disability, and Health (ICF) (53).

### Statistical Analysis

The statistical analyses were performed using IBM SPSS Statistics Version 15 (IBM, Armonk, NY). The means and standard deviations were computed to describe the continuous variables, and frequencies were used to describe the categorical variables. Chi-Square test was used to examine the difference of prevalence of neurological symptoms in subgroups. Shapiro-Wilk test was used to examine the distribution of data. Two-independent sample *t*-test (normally distributed data) or non-parametric tests (Mann-Whitney U-test for non-normally distributed data) were used to explore the differences in scores of scales and inflammatory biomarkers between the TBI patients with and without neurological symptoms, such as depression, headaches, sleep disturbance, and irritability. Bivariate correlation analysis (Pearson correlation test for continuous data and Spearman correlation test for categorical data) were conducted to explore the correlations between the inflammatory biomarkers and the neurological symptoms, and binary logistic regression analysis was used to identify the risk factors for the neurological symptoms.

## Results

### Neurological Symptoms of the TBI Patients

Of the 72 patients, nine were unable to complete the IQ test because of cognitive impairment, feelings of discomfort, refusal, and being non-communicative. The average IQ for the remaining 63 patients was 60.2 ± 9.7 (ranging from 33 to 83). The most common symptom was sleep disturbance (84.7%), followed by chronic headache (59.7%), irritability (55.6%), and depression (54.2%). The prevalence of sleep disturbance, headache, and irritability did not differ in female and male patients (*P* > 0.05). However, the prevalence of depression (85.7% vs. 46.6%) and anxiety (35.7% vs. 6.9%) in females was significantly higher than that in males (*P* = 0.008). Thirty-nine patients were categorized as depressed, and the mean PHQ score was 12.1 ± 4.0, which indicated a mild to moderate degree of depression. Notably, the chronic headache was the most common symptom complained by the patients; however, the irritability was reported by the caregivers as the most common symptom that caused greatest distress to the family members. Compared to the above symptoms, anxiety (12.5%) was less common. The global neurological disability level for the sample ranged from 4 to 10. The details are given in Table 2.

**Table 2:** Prevalence of neurological symptoms after a TBI.

**Symptoms**	**Number (%)**			X^2*^	***P***
	**Total (*n* = 72)**	**Male (*n* = 58)**	**Female (*n* = 14)**		
Depression	39 (54.2)	27 (46.6)	12 (85.7)	6.967	0.008
Sleep disturbance	61 (84.7)	50 (86.2)	11 (78.6)	0.089	0.765
Headache	43 (59.7)	32 (55.2)	11 (78.6)	2.567	0.109
Irritability	40 (55.6)	33 (56.9)	7 (50.0)	1.135	0.287
Anxiety	9 (12.5)	4 (6.9)	5 (35.7)	6.131	0.013

^*^Pearson Chi-Square test was conducted between males and females on the prevalence of each symptom.

In the patients with neurological symptoms, males scored significantly higher in chronic headache (*p* = 0.039) and irritability (*p* = 0.028) than females, which indicates more severity of these two symptoms in males. Besides, no difference was observed in the severity of other symptoms between males and females (Supplementary Table 1).

### Inflammatory Biomarkers Between the Different Groups

**Figure 1:**
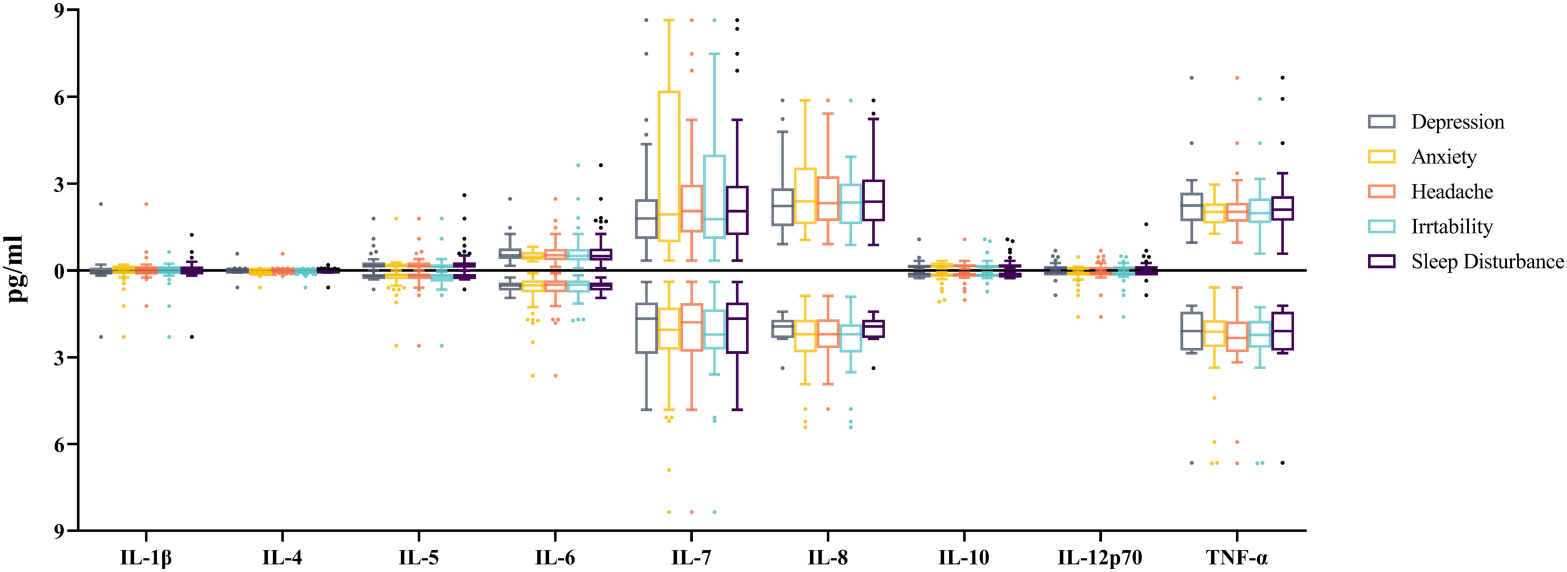
Comparisons of the cytokine levels between TBI-patients with and without related neurological symptom. 1 pg (picogram) = 10^−12^ g. The X-axis indicates the type of cytokines. The Y-axis indicates the median of the cytokine concentrations, while boxes represent lower quartile, median and upper quartile of the concentrations. Boxplots above the X-axis represent the concentrations of cytokines for patients with related neurological symptoms, while those below the X-axis represent the concentrations of cytokines for patients without related neurological symptom. The concentration of cytokines did not differ significantly between patients with and without each neurological symptom (All *P* > 0.05).

According to the result of Shapiro-Wilk test, the concentrations of inflammatory biomarkers were non-normally distributed (*P* < 0.05). The Mann-Whitney U test showed no male and female differences for all the investigated inflammatory biomarkers (Supplementary Table 2), and no inflammatory biomarker differences were found between the TBI patients with and without the investigated neurological symptoms (Figure 1).

### Correlation Analysis Between Demographic, Neurological Symptoms, and Inflammatory Biomarkers

The results of the bivariate Spearman correlation analysis showed that age was positively associated with IL-5 (r = 0.306, *P* = 0.009), TNF-α (r = 0.350, *P* = 0.003), IL-6 (r = 0.263, *P* = 0.025), sleep disturbance (r = 0.257, *P* = 0.029), and depression (r = 0.340, *P* = 0.004), but negatively associated with irritability (r = −0.321, *P* = 0.006). Considering the effect of age on the symptoms and biomarkers, a partial correlation analysis was conducted to evaluate the associations between the inflammatory biomarkers and the neurological symptoms independent of age. Of the neurological symptoms, chronic headache was positively associated with both depression (r = 0.251, *P* = 0.035) and irritability (r = 0.250, *P* = 0.036). Inflammatory biomarkers IL-5, IL-6, and IL-8 were positively associated with sleep disturbance (r = 0.242, *P* = 0.041; r = 0.309, *P* = 0.008; r = 0.236, *P* = 0.046); however, only the association with IL-6 persisted after adjusting the effect of age. The bivariate correlation analysis also revealed that IL-6 was positively associated with depression (r = 0.275, *P* = 0.019); however, the association was absent after controlling for the age effect. IL-5 and IL12p70 were negatively associated with WAIS IQ (r = −0.237, *P* = 0.047; r = −0.260, *P* = 0.028) and the global neurological disability level (r = −0.325, *P* = 0.006; r = −0.319, *P* = 0.007) even after adjusting for the age effect. Details are presented in Table 3.

**Table 3:** Correlation analysis of inflammatory biomarkers with psychiatric symptoms.

	**IL-1β**	**IL-4**	**IL-5**	**IL-6**	**IL-7**	**IL-8**	**IL-10**	**IL-12p70**	**TNF-α**
Age	0.074 (0.537)	0.045 (0.709)	0.302 (0.010)*	0.196 (0.098)	−0.026 (0.828)	0.164 (0.168)	0.087 (0.469)	0.062 (0.603)	0.259 (0.028)*
Depression	−0.028 (0.818)	0.081 (0.500)	0.111 (0.354)	0.048 (0.686)	−0.094 (0.430)	−0.080 (0.502)	0.019 (0.874)	−0.056 (0.638)	0.091 (0.449)
Chronic headache	−0.052 (0.663)	−0.005 (0.964)	−0.045 (0.710)	0.082 (0.491)	0.089 (0.457)	−0.027 (0.820)	−0.042 (0.728)	−0.043 (0.722)	−0.110 (0.358)
Sleep disturbance	−0.008 (0.818)	−0.046 (0.703)	0.242 (0.041)* 0.105 (0.126)^#^	0.309 (0.008)* 0.274 (0.021)#	0.135 (0.258)	0.236 (0.046)* −0.098 (0.139)#	0.159 (0.182)	0.120 (0.313)	0.136 (0.225)
Irritability	−0.016 (0.376)	−0.090 (0.111)	−0.192 (0.105)	0.061 (0.613)	0.046 (0.702)	0.123 (0.304)	0.041 (0.732)	−0.172 (0.147)	−0.103 (0.390)
Psychiatric disability	−0.131 (0.273)	−0.109 (0.361)	−0.295 (0.012)*−0.325 (0.006)#	−0.028 (0.817)	−0.063 (0.598)	−0.026 (0.825)	−0.108 (0.366)	−0.315 (0.007)* −0.319 (0.007)#	−0.143 (0.230)
WAIS IQ	−0.031 (0.798)	−0.053 (0.660)	−0.229 (0.045)*−0.237 (0.047)#	0.038 (0.751)	−0.040 (0.741)	0.023 (0.850)	0.107 (0.372)	−0.260 (0.027)* −0.260 (0.028)#	−0.022 (0.853)
Days post injury	−0.118 (0.327)	−0.147 (0.222)	−0.060 (0.620)	−0.042 (0.726)	−0.134 (0.264)	0.087 (0.468)	−0.215 (0.072)	−0.171 (0.155)	−0.184 (0.105)

Numbers outside the bracket are r values for the correlations; numbers in the bracket are P-values, while the P-values <0.05 was marked by*. ^#^Is the r and P-values for the correlations after adjusting the effect of age.

### Binary Logistic Regression Analysis

To investigate the risk factors for the neurological symptoms, a binary logistic regression analysis was conducted on the participants with and without specific headache, sleep disturbance, depression, and irritability symptoms. TNF-α was identified as a protective factor for post-TBI chronic headache (OR = 0.473). IQ (OR = 1.041) and headache (OR = 1.024) was a risk factor for depression. Age was a protective factor for irritability (OR = 0.941) and headache was a risk factor (OR = 1.018) (Table 4). More information about the regression results is presented in Supplementary Table 3.

**Table 4:** Risk and protective factors for neuropsychiatric symptoms post-TBI identified from the binary logistic regression.

	**OR**	**95% CI**	***p***
**Headache**			
TNF-α	0.473	0.235–0.952	0.036
**Depression**			
IQ	1.041	1.006–1.045	0.024
Headache	1.024	1.003–1.045	0.024
**Irritability**			
Age	0.941	0.897–0.987	0.012
Headache	1.018	1.000–1.036	0.050

## Discussion

This study investigated the serum concentrations of nine inflammatory biomarkers in 72 TBI patients in the post-TBI chronic phase (from 6 months to 2 years) and analyzed the association of these biomarkers with neurological symptoms including depression, chronic headache, sleep disturbance, irritability, anxiety, WAIS IQ, and global neuropsychiatric disability.

### Sleep Disturbances

Of the 72 TBI patients, the most common symptom was sleep disturbance (84.7%), followed by chronic headache (59.7%), irritability (55.6%), and depression (54.2%). The high prevalence of post-TBI sleep disturbance was in line with the results of a previous review study that reported the sleep disturbances were the most common TBI patient complaints that contributed to morbidity and long-term sequelae (54). A meta-analysis comprising 21 studies reported that about 50% of TBI patients suffered from sleep-wake disturbances (55), and Makley et al. found that up to 68% of patients had sleep disturbances in TBI rehabilitation units (56).

The current study also found that IL-5, IL-6, and IL-8 were positively associated with sleep disturbance; however, only the association with IL-6 persisted after controlling for age, which indicated that a higher circulation IL-6 level was related to more severity of sleep disturbance. Although the association has no causality property, a bidirectional relationship between sleep and inflammation has been verified in TBI patients. IL-6 has been recognized as one of the most important biomarkers for chronic or systemic low-grade inflammation and has the most non-immunological effects on tissues (57). Malik et al. noted in a review study that IL-6 was one of the most common cytokines associated with poor psychological outcomes in a chronic mTBI population (23). Specifically, it has been proposed that IL-6 is the “sleep factor” that mediates sleep continuity and sleep macrostructure (58, 59). Several studies have reported that sleep disturbances can increase systemic IL-6 concentrations (60, 61). For example, Nemeth et al. reported that a reduction in sleep duration and quality led to a significant elevation in IL-6 serum levels (61). Preliminary evidence has also suggested that IL-6 may modify circadian rhythms by activating period gene 1 (Per 1) transcription (62). Furthermore, another study found that sleep can modify immune system functions by inducing changes in the hypothalamus-pituitary-adrenal axis and the sympathetic nervous system (63).

In addition to the aforementioned studies based on non-TBI population, Gottshall et al. also investigated the association between sleep quality and cytokines (IL-10, IL-6, and TNF-α) in the plasma and plasma-derived extracellular vesicles (EVs) in chronic mTBI (64). Same to the current study, they also found that plasma IL-6 was positively correlated with sleep quality; however, this association was absent in their study after adjusting for the effect of age, sex, and BMI. The difference in participants may account for this different result. Gottshall et al. examined the US military servicemembers and veterans who had lifetime mTBI history with an average of 9 years interval post injury; however, the current study was based on Chinese population who suffered moderate to severe TBI 6 months to 2 years ago (the average interval was 334 ± 163 days). In addition, the difference in the age of the study population (the mean age was 40 years in Gottshall et al.'s study and 48 years in the current study) may also account for the difference results. Future studies based on larger sample with higher homogeneity are warranted in verifying the association between IL-6 and sleep quality in TBI patients. In addition, as TBI patients face long-term psychological stress, this may be a critical factor for chronic inflammation and sleep disturbance. Therefore, the interactions between inflammation, the HPA-axis, and sleep disturbance post TBI is worthy of further study. Sleep disturbances after a TBI, particularly in the chronic phase, contribute to an exacerbation of neurocognitive and neurobehavioral deficits and prolong the recovery phase after the injury (65, 66). Therefore, early recognition and intervention may limit the secondary negative TBI effects and improve patient outcomes (67).

### Chronic Headache

Chronic headache was the second most common symptoms in the post-TBI chronic phase, with a prevalence of 59.7%. In line with this, a review study that examined data from more than 23 studies covering 4,200 TBI patients found that 51.5% of TBI patients experienced chronic pain (68). In the current study, persistent headache was the most common symptom complained by the patients, which was reported to significantly influence their daily lives and cause extreme emotional distress. Furthermore, chronic headache was identified as a significant risk factor both for depression and irritability, which highlights the contribution chronic headache makes to emotional disturbance and related cognitive-behavioral problems. This study highlighted that chronic headache may be a mediator for emotional and behavioral problems in TBI patients and should be an important target for post-TBI neurological symptom interventions. In further, the interactions between chronic headache, depression, irritability, and sleep disturbance post-TBI should receive more attention (54).

Although chronic headache is common and appears to play a crucial role in TBI-related disabilities, the exact mechanisms are still unclear. Neuroinflammation has been reported as an important mechanism for chronic headache (12, 14, 69). For example, Irvine et al. found that several neuroinflammation and pain-related cytokines such as TNF-α and IL-1βincreased in the thalamus, hippocampus, substantia nigra, and hypothalamus of TBI patients (12). Aydin et al. reported the serum TNF-α was significant higher in patients with migraine with aura during migraine attacks (70). Perini et al. found that circulating TNF-α levels were higher in patients with migraine studied soon after attacks onset and lower over time (71). The difference in the direction of the association between cytokines and headaches are varied in different studies, because of the difference in the study population, the type of headaches and the time interval between the headache attacks and the sample collection. In the current study, TNF-α was found to be a protective factor for post-TBI chronic headache, which was supported by a number of previous studies. Previous studies have shown that in addition to its pro-inflammatory activity, TNF-α also acts as a neurotrophic factor that is vital for neuroprotection and repair (72, 73). Genetic studies have also found that the function of TNF-α differs in the acute and delayed post-TBI phases. Immediately after the injury, TNF-α seems to act as a potent inflammatory mediator; however, later it has been found to be the neurotrophic factor required for neuroprotection and repair (74). Another study found that when the TNF-α receptor gene was knocked out, post-TBI mice had exacerbated tissue and blood brain barrier (BBB) damage and neurological impairment, suggesting that TNF-α may have neuroprotective effects (75).

### Depression and Irritability

Of the 72 TBI participants, 54.2% reported depression in the chronic post-TBI phase. This prevalence was similar to Bombardier et al., which reported that 53.1% of 559 TBI patients met the criteria for a major depressive disorder (MDD) in the first year after the TBI (76). Major depression has been reported to be the most prevalent neuropsychiatric disorder following a TBI, with a period prevalence of 33% to 42% within the first year and 61% in the first 7 years following the injury (77). Review study has reported that the best current evidence suggests that depressed mood in persons with a TBI is characterized more by irritability, anger, and aggression than by sadness and tearfulness (78), which was also verified in the current study. In the current study, irritability was reported as the most common emotional problem that caused the most distressing burden to caregivers. In line with this, Kersel et al. also reported that emotional control problems were the most distressing issue for patients and caregivers at 1 year post-injury in a cohort of severe TBI patients (79). Therefore, neurological rehabilitation for TBI patients should focus greater attention on psychological and neurological management and treatment for irritability.

There are many potential mechanisms to explain post-TBI depression and the related emotional dysregulation, such as brain area and related circuit damage (e.g., pre-frontal, hippocampus, and frontal lobe-basal ganglia circuit) (80), neuroinflammation, axonal degeneration, and psychosocial factors (disruption to family and social relationships, functional impairment, loneliness, loss of employment, and financial worries) (81). An interesting finding in the current study was that IQ was identified as a risk factor for depression. One explanation for this could be that patients with higher IQs have a greater focus on their future, and therefore feel more depressed when confronted with their negative TBI-related consequences. On the contrary, patients with lower IQ may have more severe executive ability impairments and would possibly not pay attention to thinking about their future life and the psychosocial factors. This result highlights that psychosocial factors may play crucial role in the development of depression in TBI patients; therefore, greater attention should be focused on TBI patients with higher IQs and executive functions in preventing depression post TBI.

Some studies have documented an association between inflammation and depression and reported that higher concentrations of pro-inflammatory cytokines possibly directly contribute to the development of depressive symptoms (11, 82, 83). The current study found that the serum IL-6 level was positively associated with depression; however, this association was not significant after controlling for the age effect. Age is positively associated with depression and IL-6, which indicates that older TBI patients are more likely to develop post-injury depression and IL-6 may have a greater impact in older populations. Given the relatively small sample size of the current study, further studies with larger sample sizes and with more subgroups are needed.

### Neurological Disability

IL-5 and IL12p70 were negatively associated with WAIS IQ and the global neurological disability level of the TBI patients, which suggested that IL-5 and IL-12p70 were associated with the global neurological function in the chronic post-TBI phase. IL-12p70 is the active form of IL-12, which is an important type 1 immune cytokines and is mainly produced by macrophages and dendritic cells (84). Several studies have concluded that IL-12p70 is associated with cognitive impairment and neurodegenerative disorders such as Alzheimer's disease (AD) because of its role in the neuro-inflammatory processes (85, 86). Trollor et al. reported that IL-12 contributed to cognitive decline such as reduced processing speed performances in elderly individuals aged 70–90 years in an Australian population (87), and older individuals with mild cognitive impairment have also been found to have higher levels of IL-12 (88). A study in Taiwan also found that the IL12 receptor (β2) gene played a vital role in modulating cognitive aging (89). A meta-analysis study also found significantly higher levels of IL-12 in peripheral blood in AD patients compared with the healthy controls (90). Additionally, a recent study used a machine learning prediction model to assess 242 blood proteins in 80 older adults and found that IL-12 was one of the three proteins that could predict cognitive impairment (91). Papenberg et al. also found that IL-12 was associated with Mini-Mental State Examination (MMSE) test scores and the gray-matter volumes in the lateral prefrontal cortex and hippocampus in older adults (92).

The IL-12 was simultaneously associated with the global neurological disability in the chronic phase after TBI, and the neurogenerative deficits in elder adults, indicating that there may be some common pathogenesis for TBI and elder neurodegenerative deficits. Accumulated epidemiological studies have identified TBI as a risk factor for delayed neurodegenerative disorders including Alzheimer's disease, dementia and chronic traumatic encephalopathy (93–95). However, the underpin pathogenesis for this link is still not clear. Given the emerging studies reported that neuroinflammation may persist for decades after TBI, it may play a critical role in the link between TBI and the delayed neurodegenerative disorders. Preclinical studies have demonstrated that persistent neuroinflammation characterized by microglia activation would reduce neurogenesis and synaptogenesis, and further to reduce cortical dendritic complexity and neuronal connectivity (30, 96, 97), which would contribute to neurological deficits and on-going neurodegenerative processes after TBI (98–100). Moreover, animal studies have showed that microglial inhibition or depletion during chronic phase of experimental TBI could reduce neurodegeneration and neurological deficits, such as cognitive and memory impairments (98, 101), which indicates that neuroinflammation may also serve as a therapeutic target for the rehabilitation in TBI patients. The current study only examined the serum levels of cytokines, and did not investigate the cytokine levels in brain areas. Given that the cytokines could travel across disrupted Blood Brain Barrier (BBB) after TBI (102), future studies could simultaneously measure cytokines in brain areas and blood so as to provide more information about the inflammatory mechanisms of the long-term neurological deficits after TBI.

### Limitations and Future Directions

There were some weaknesses in the current study that need to be considered. First, the sample size was relatively small, which may have limited the statistical ability to detect small differences between the groups. The current study was a preliminary exploration of the association between inflammatory biomarkers and neurological symptoms in the chronic phase of TBI patients. Based on the preliminary evidence for this association, future studies with larger sample sizes are warranted. Second, this study only investigated nine inflammatory markers. further studies are needed to assess a broader array of markers involving oxidative stress and HPA axis, from which a more general and informative picture of the pathogenesis of TBI-related neurological symptoms and the inflammatory mechanisms could be obtained. Third, the current study only compared the inflammation in different TBI subgroups with and without specific neurological symptoms, and investigated the association of inflammatory cytokines with different neurological symptoms. Future studies could include healthy controls and depressive patients without TBI as comparison subgroups, by which we could get a more comprehensive understanding of the inflammation profiles for post-TBI neurological symptoms. Fourth, the menstrual status of female participants was not collected in the current study. Hormonal levels of women in different menstrual status may affect mental and behavioral symptoms and may affect the levels of biomarkers of inflammation, which should be considered as an influencing factor when investigating the association between inflammatory biomarkers and psychiatric symptoms in the future. Last but not least, body mass index (BMI) has been reported to be positively associated with the level of inflammatory biomarkers such as CRP and IL-6 (103), which should be included as a factor in the future studies on inflammation.

## Conclusion

Inflammation is a shared neurophysiologic underpinning for TBI and various neurological symptoms, which provides a promising framework to investigate the mechanisms of the persistent neurological disabilities following a TBI. However, few studies have focused on the association between chronic inflammation and neurological symptoms in TBI patients. This is one of the few studies that has focused on chronic cytokine profiles and their relationships with neurological symptoms post-TBI. The results showed that inflammation cytokines were related to sleep disturbance, headaches, and the global neurological function in the post-TBI chronic phase, which adds evidence to the reciprocal relationship between inflammation and neurological symptoms following a TBI. The results highlighted the potential role of anti-inflammatory for the TBI patient's neurological function rehabilitation. The present study is just a preliminary investigation of the association between inflammation and neurological symptoms following a TBI. Given the small sample size and relatively small correlation coefficients, further research with larger sample sizes and more related biomarkers are needed to more thoroughly elucidate this association and the underlying mechanisms.

## Data Availability Statement

The original contributions presented in the study are included in the article/Supplementary Material, further inquiries can be directed to the corresponding author/s.

## Ethics Statement

The studies involving human participants were reviewed and approved by Medical Ethical Committee of Sichuan University. The patients/participants provided their written informed consent to participate in this study.

## Author Contributions

GL, YY, and JH: conceptualization. GL: data analysis, funding acquisition, and writing-original draft. GL, HL, YH, ZH, YL, YG, and JH: data collection and experiments. YY and JH: methodology, supervision, writing-review, and editing. All authors have read and agreed to the published version of the manuscript.

## Funding

This work was supported by the China Postdoctoral Science Foundation (2018M643488) and the National Natural Science Foundation of China (81901928).

## Conflict of Interest

The authors declare that the research was conducted in the absence of any commercial or financial relationships that could be construed as a potential conflict of interest.

## Publisher's Note

All claims expressed in this article are solely those of the authors and do not necessarily represent those of their affiliated organizations, or those of the publisher, the editors and the reviewers. Any product that may be evaluated in this article, or claim that may be made by its manufacturer, is not guaranteed or endorsed by the publisher.
