# Supplementary material for: Neurological Symptoms and Their Associations With Inflammatory Biomarkers in the Chronic Phase Following Traumatic Brain Injuries
Source: Front Psychiatry. 2022 Jun 24;13:895852. doi: 10.3389/fpsyt.2022.895852 (PMC9263586; doi:10.3389/fpsyt.2022.895852)
Supplement: Supplementary file 1 [file Data_Sheet_1.docx]

**Table 1.** Comparison of mean scores for each neuropsychiatric symptom between males and females with neuropsychiatric symptoms

| Variables | Male | |  | Female | | *P* |
| --- | --- | --- | --- | --- | --- | --- |
|  | n* | Mean(SD) |  | n* | Mean(SD) |  |
| Age | 58 | 46.59(12.445) |  | 14 | 52.50(9.045) | 0.195 |
| IQ | 58 | 52.43(23.178) |  | 14 | 53.86(17.119) | 0.593 |
| Depression | 27 | 11.815(3.923) |  | 12 | 12.583(4.420) | 0.464 |
| Anxiety | 4 | 18.333(2.877) |  | 5 | 20.667(3.055) | 0.129 |
| Headache | 32 | 42.188(26.969） |  | 11 | 57.273(24.532） | 0.039 |
| Irritability | 33 | 6.212(0.992) |  | 7 | 5.268(2.43) | 0.028 |
| Sleep Disorder | 50 | 14.200(4.199） |  | 11 | 12.730(4.338） | 0.265 |

Note. *n is the sample size for each subgroup. The mean score is for the patients who were recognized as having the index symptoms as the scale scores were equal to or above the cut-off score.

**Table 2.** Comparison of biomarker concentrations between males and females

| Biomarkers | Male(median)^#^ | Female(median)^#^ | U* | *P* |
| --- | --- | --- | --- | --- |
| IL-1β | 0.071 | 0.054 | 293.000 | 0.098 |
| IL4 | 0.012 | 0.009 | 339.500 | 0.344 |
| IL5 | 0.168 | 0.149 | 399.00 | 0.921 |
| IL6 | 0.493 | 0.670 | 498.500 | 0.188 |
| IL7 | 2.050 | 1.833 | 416.500 | 0.881 |
| IL8 | 2.208 | 2.278 | 356.000 | 0.477 |
| IL10 | 0.153 | 0.116 | 313.500 | 0.188 |
| IL12p70 | 0.096 | 0.063 | 301.500 | 0.137 |
| TNF-a | 2.051 | 2.243 | 433.500 | 0.696 |

Note. *Mann-Whitney U test. ^#^Numbers are medians of the inflammatory biomarkers.

**Table 3.** Risk and protective factors for neuropsychiatric symptoms post-TBI identified from the binary logistic regression

|  | OR | 95% CI | *p* |
| --- | --- | --- | --- |
| Headache | | | |
| TNF-α | 0.473 | 0.235-0.952 | 0.036 |
| IL-6 | 0.711 | 0.203-2.487 | 0.594 |
| IL-10 | 1.647 | 0.031-87.085 | 0.805 |
| IL-5 | 0.593 | 0.051-6.884 | 0.676 |
| IL-7 | 1.300 | 0.884-1.909 | 0.182 |
| IL-8 | 1.047 | 0.753-1.455 | 0.786 |
| IL-12p70 | 0.327 | 0.003-35.015 | 0.640 |
| Gender | 2.143 | 0.424-10.832 | 0.357 |
| Age | 1.008 | 0.959-1.060 | 0.741 |
| IQ | 0.988 | 0.959-1.018 | 0.418 |
| Depression | 1.074 | 0.972-1.188 | 0.162 |
| Depression | | | |
| IQ | 1.041 | 1.006-1.045 | 0.024 |
| Headache | 1.024 | 1.003-1.045 | 0.024 |
| IL-10 | 0.117 | 0.003-1.573 | 0.095 |
| Anxiety | 1.121 | 0.985-1.276 | 0.085 |
| Sleep disturbance | 0.943 | 0.410-2.169 | 0.891 |
| Irritability | 0.893 | 0.745-1.069 | 0.893 |
| TNF-α | 1.540 | 0.833-2.846 | 0.168 |
| IL-6 | 1.141 | 0.369-3.530 | 0.819 |
| IL-7 | 0.725 | 0.639-1.366 | 0.934 |
| IL-8 | 1.625 | 0.264-10.006 | 0.601 |
| IL-1β | 0.002 | 0.000-1.273 | 0.059 |
| Irritability | | | |
| Age | 0.941 | 0.897-0.987 | 0.012 |
| Headache | 1.018 | 1.000-1.036 | 0.050 |
| IL-5 | 1.042 | 0.072-15.077 | 0.976 |
| IL-6 | 1.996 | 0.359-11.081 | 0.430 |
| IL-7 | 0.912 | 0.620-1.342 | 0.640 |
| IL-8 | 0.983 | 0.661-1.463 | 0.932 |
| IL-12p70 | 0.226 | 0.001-47.994 | 0.586 |
| Gender | 0.213 | 0.032-1.430 | 0.111 |
| IQ | 0.991 | 0.958-1.026 | 0.620 |
